# Highly neutralizing anti–IFN-ω autoantibodies underlie severe pediatric adenovirus infection

**DOI:** 10.70962/jhi.20250255

**Published:** 2026-10-13

**Authors:** Chen-Xuan Kang, Jing-Ya Ding, Yu-Fang Lo, Tsai-Yi Wu, Han-Po Shih, Jhan-Jie Peng, Kung-Wei Lin, You-Ning Lin, Chia-Chi Lo, Shao-Hsuan Hsia, Jainn-Jim Lin, En-Pei Lee, Oi-Wa Chan, Yhu-Chering Huang, Chih-Jung Chen, Tzou-Yien Lin, Cheng-Lung Ku, Chen-Yen Kuo

**Affiliations:** 1 Laboratory of Human Immunology and Infectious Diseases, Institute of Immunology and Translational Medicine, Chang Gung University, Taoyuan, Taiwan; 2 Graduate Institute of Clinical Medical Sciences, Chang Gung University, Taoyuan, Taiwan; 3 Center for Molecular and Clinical Immunology, Chang Gung University, Taoyuan, Taiwan; 4 Division of General Surgery, Department of Surgery, Chang Gung Memorial Hospital, Taoyuan, Taiwan; 5 Institute of Microbiology and Immunology, School of Life Sciences, National Yang Ming Chiao Tung University, Taipei, Taiwan; 6 Physician Assistant Department, University of Maryland Eastern Shore, Princess Anne, MD, USA; 7 Division of Pediatric Critical Care Medicine, Chang Gung Memorial Hospital, Taoyuan, Taiwan; 8 Division of Infectious Diseases, Department of Pediatrics, Chang Gung Memorial Hospital, Taoyuan, Taiwan; 9 College of Medicine, Chang Gung University, Taoyuan, Taiwan; 10 Center for Drug Research and Development, College of Human Ecology, Chang Gung University of Science and Technology, Taoyuan, Taiwan; 11 Department of Chinese Medicine, Chang Gung University, Taoyuan, Taiwan

## Abstract

Neutralizing autoantibodies (auto-Abs) against type I IFNs (IFN-α and IFN-ω) have emerged as major acquired risk factors for severe viral infections. Human adenovirus (HAdV) is highly prevalent in children and can cause severe pneumonia, but the host determinants of disease severity remain poorly understood. We describe here a 5-year-old child with life-threatening HAdV-B7 pneumonia requiring intensive care who carried high-titer anti–IFN-ω auto-Abs of the IgG1 and IgG3 subclasses. Peripheral blood mononuclear cells produced normal amounts of IFN-α and IFN-ω ex vivo; however, IFN-ω was undetectable in the plasma during acute illness. Functional assays demonstrated that the patient’s plasma strongly neutralized IFN-ω, abolishing its antiviral signaling and protective effects, resulting in high levels of adenovirus replication and virus-induced cytotoxicity. These findings implicate anti–IFN-ω auto-Abs in severe adenoviral infection and support immunological screening of pediatric patients with unexplained severe viral pneumonia, which may guide targeted management.

## Teaser

A case of neutralizing anti–IFN-ω autoantibodies impairing antiviral defense and driving severe adenovirus pneumonia in a child.

## Graphical Abstract

**Figure:**
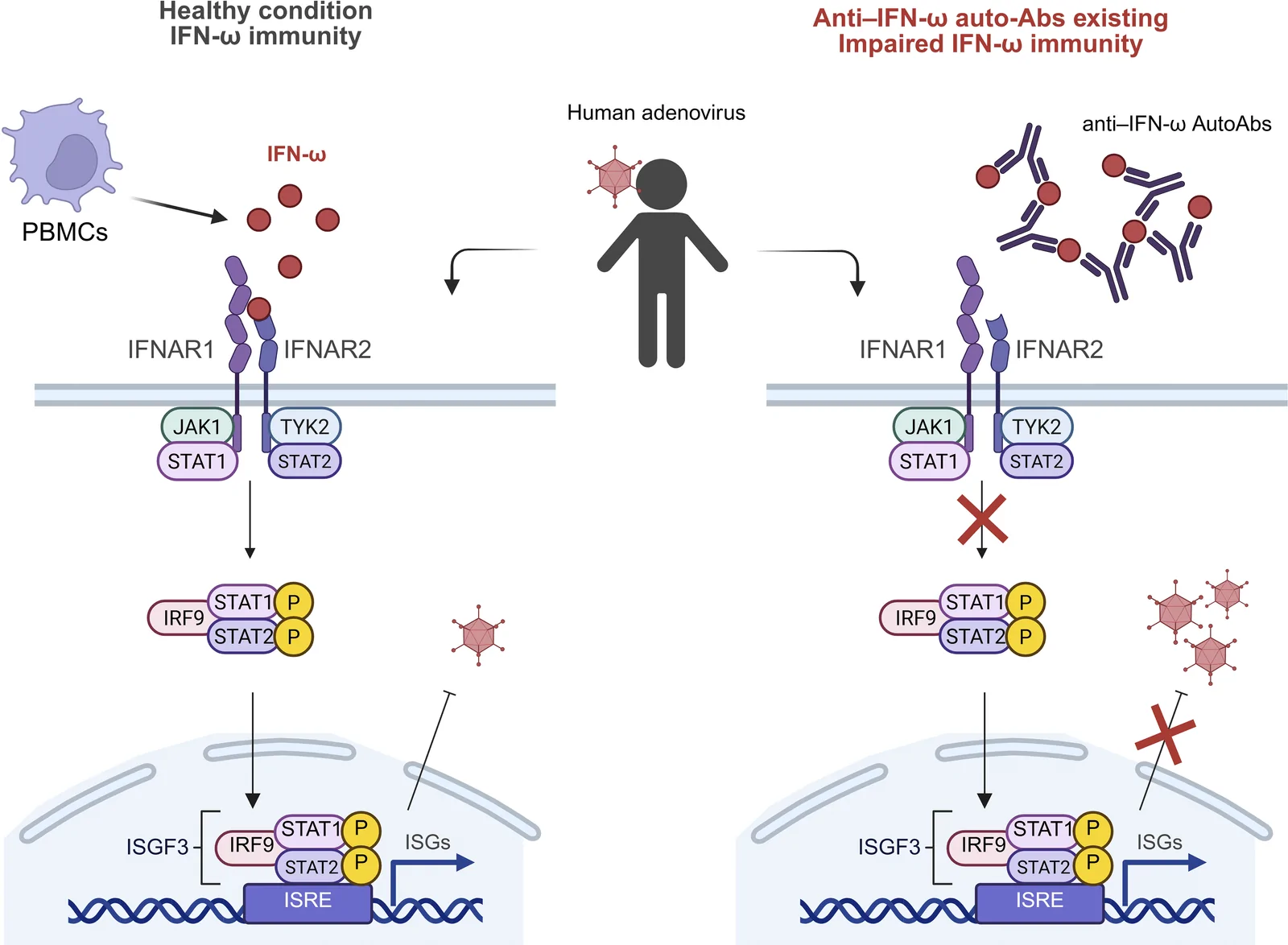

## Introduction

Human adenoviruses (HAdVs) are non-enveloped double-stranded DNA viruses that commonly infect children under 5 years of age, largely due to the immaturity of humoral immunity in young children (1, 2). HAdVs are classified into seven species (A–G) comprising 113 genotypes (http://hadvwg.gmu.edu). HAdV infections can manifest in diverse clinical forms, respiratory disease being the most common, accounting for approximately 2–5% of all respiratory infections worldwide (3, 4). HAdVs are also implicated in gastroenteritis, conjunctivitis, and hemorrhagic cystitis (1, 2, 5). HAdV species B7 (HAdV-B7) is a major cause of lower respiratory tract infections, particularly pneumonia. Numerous outbreaks and sporadic cases of HAdV-B7–associated pneumonia have been reported globally, highlighting the widespread clinical impact of this condition (3, 6, 7). In Taiwan, community outbreaks were documented in 1999 and 2011, and subsequent retrospective studies confirmed that the virus was continuing to circulate (8, 9, 10). Most HAdV-B7 infections in immunocompetent individuals are mild and self-limiting, but a subset—affecting immunocompromised hosts, such as transplant recipients or patients with underlying immunodeficiency, in particular—progress to severe or disseminated disease (11, 12, 13). Rare cases of severe HAdV-B7 pneumonia have also occurred in otherwise healthy individuals (14). Elucidating the host factors underlying predisposition to severe HAdV-B7 infection therefore remains a critical unmet need, as the mechanisms driving its pathogenesis in natural infection remain poorly understood.

Type I IFNs (IFNs-I)—encompassing 13 IFN-α subtypes, and one subtype each of IFN-β, IFN-ω, IFN-ε, and IFN-κ—play key roles in orchestrating innate and adaptive immune responses to viral infection (15, 16, 17). They signal through the IFNAR1/IFNAR2 receptor complex and share overlapping antiviral mechanisms (18). IFN-ω, first identified in 1985, has 62 and 33% amino acid sequence identity to IFN-α and IFN-β, respectively (19). It has diverse biological effects, including potent antiviral, immunomodulatory, and antitumor activities (20, 21). IFN-ω displays strong antiviral activity in vitro and has been approved for veterinary applications (22, 23, 24, 25, 26), but its physiological role in humans remains incompletely defined. Deficient IFN-I responses—whether due to inborn errors of IFN-I immunity (27, 28, 29) or the presence of neutralizing autoantibodies (auto-Abs) against IFN-I (AAN-I-IFNs)—have been implicated in life-threatening viral infections. For example, in COVID-19, more than 15% of patients with critical pneumonia harbor preexisting neutralizing auto-Abs against IFN-α and/or IFN-ω (30). Similar associations have been reported in severe adverse reactions to the yellow fever vaccine (∼30% of cases), critical influenza pneumonia (∼5%), severe Middle East respiratory syndrome (∼25%), and viral encephalitis caused by West Nile virus (∼40%) (31, 32, 33, 34). Further investigations are therefore warranted to delineate the mechanisms by which auto-Abs against IFN-α and/or IFN-ω contribute to the pathogenesis of severe viral infections.

In this study, we identified anti–IFN-ω auto-Abs in a 5-year-old boy with severe HAdV-B7 pneumonia. We characterized these auto-Abs, evaluated the antiviral role of IFN-ω, and determined whether plasma containing anti–IFN-ω auto-Abs could impair IFN-ω–mediated antiviral protection during HAdV-B7 infection.

## Results

### Case report

**Figure 1.**
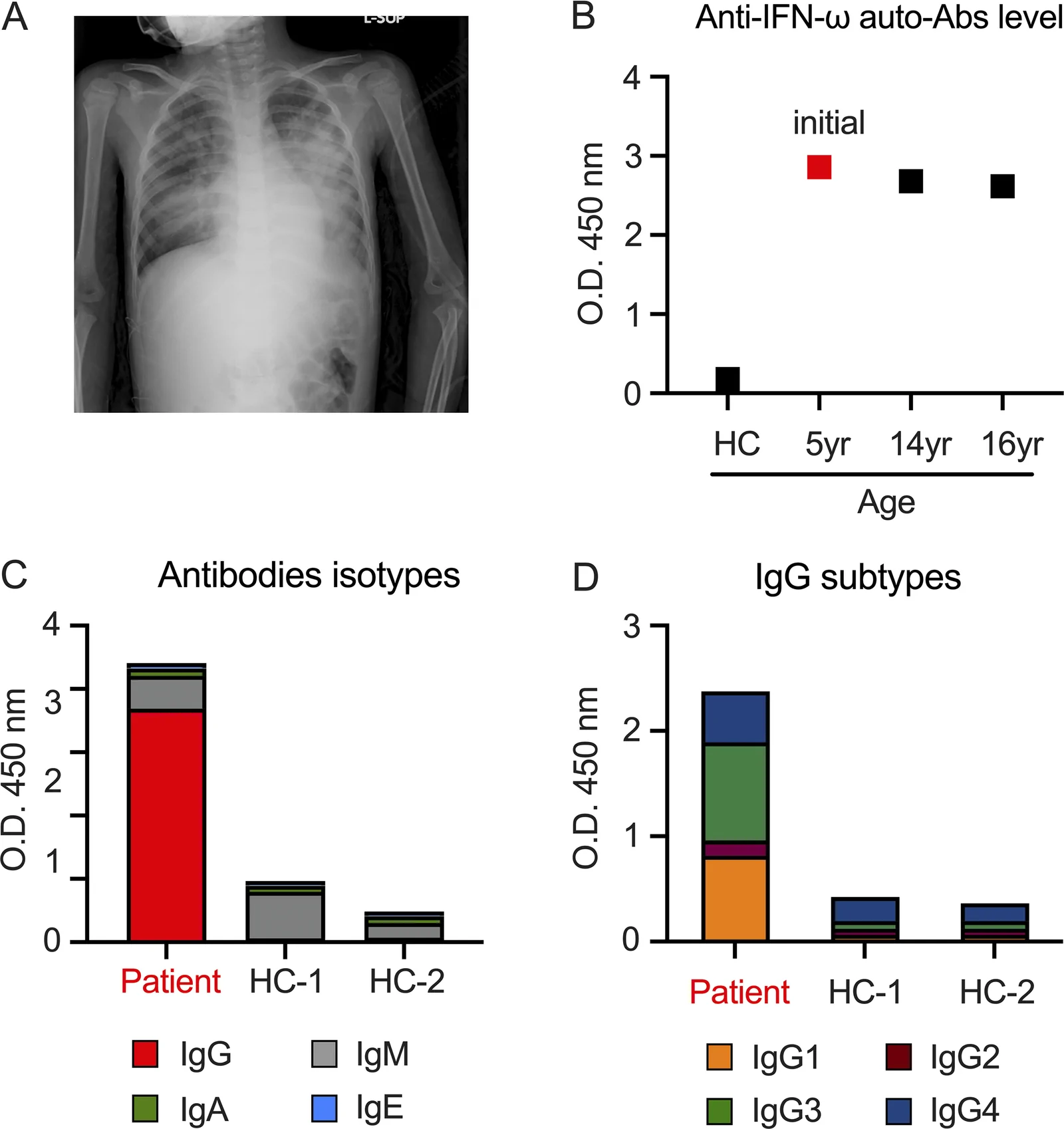
**Detection and characterization of anti–IFN-ω auto-Abs in a child with severe adenoviral pneumonia. (A)** Chest X-ray of the patient with severe pneumonia with bilateral consolidation. **(B)** Plasma anti–IFN-ω auto-Ab levels during the acute phase (initial) and after infection. **(C and D)** Ig isotypes and IgG subclasses of anti–IFN-ω auto-Abs in the patient’s plasma, as determined by ELISA. Plasma samples were analyzed at a dilution of 1:100. HC, healthy control. The three patient plasma samples were collected during the acute phase of infection and at follow-up, 9 and 11 years (yr) after the initial infection.

A previously healthy boy was referred, at the age of 5 years and 6 mo, from a district hospital to the pediatric intensive care unit of Chang Gung Memorial Hospital in 2011 for progressive dyspnea following a 9-day history of high fever and cough. Upon admission, his vital signs were checked. He had a body temperature of 38.8°C, a respiratory rate of 38 breaths per minute, a heart rate of 138 beats per minute, and blood pressure of 115/67 mmHg. Physical examination revealed subcostal and intercostal retractions, tachypnea, tachycardia, and bilateral crackles and wheezing on auscultation. Laboratory investigations demonstrated anemia (hemoglobin, 10.8 g/dl), markedly high C-reactive protein (CRP) (67.4 mg/L), creatine phosphokinase (4,365 U/L) levels, and high levels of liver enzymes (AST, 179 U/L; ALT, 43 U/L). Chest X-ray showed patchy opacity in the left lower lobe and considerable bilateral parahilar-peribronchial infiltration (Fig. 1 A). Based on these clinical and radiological findings, a presumptive diagnosis of sepsis with severe pneumonia was made. Supplemental oxygen therapy via mask was initiated at a fraction of inspired oxygen of 40%, together with intravenous vancomycin and ceftriaxone, and nebulized terbutaline. A rapid antigen test for HAdV performed on a throat swab was positive on day 1 of hospitalization. Despite initial management, the patient’s respiratory condition worsened. On day 3 of hospitalization, a follow-up chest X-ray revealed a progression of pneumonia, with an increase in the number and size of the patchy opacities in the left upper, left lower, and right lower lobes. Budesonide nebulization was introduced on day 4, in response to ongoing respiratory distress. The patient became afebrile by day 6 and remained so thereafter. Viral cultures from throat and rectal swabs subsequently revealed the presence of adenovirus type 7. Sputum and blood cultures yielded no bacterial growth. Multiplex real-time PCR on serial plasma samples obtained from days 1–7 of hospitalization showed a progressive rise in adenovirus DNA levels, indicating systemic dissemination. No evidence of other viral infections was detected in these samples. The patient required oxygen support to maintain peripheral oxygen saturation between 90 and 100% until day 9 of hospitalization. Despite respiratory distress, he remained hemodynamically stable throughout hospitalization, without the need for vasoactive agents. His clinical condition gradually improved, and he was discharged after 12 days of hospitalization.

The patient had no notable birth, personal, or family medical history. There were no prior episodes of recurrent respiratory infections or known congenital or acquired cardiac conditions. Immunological evaluation during the acute phase revealed marked lymphopenia, whereas the relative distribution of lymphocyte subsets and Ig levels were largely preserved (Table 1). Whole-exome sequencing revealed no pathogenic variants in genes associated with inborn errors of immunity (35). The patient’s plasma sample was collected 6 days after admission.

**Table 1.** Laboratory findings during the acute phase of adenoviral infection

Hematology	Value	Reference range
Hb (g/dl)	10.8	11.6–13.7
RBC (10^6/μl)	4.01	4.28–5.05
Ht (%)	30.1	34.2–39.8
PLT (10^3/μl)	234	150–400
WBC (/μl)	4,600	6,000–10,400
CRP (mg/L)	67.4	<5

WBC-DC (%)	Value	Reference range (%)
Band	7	0–3
Seg	81	27.8–57.6
Eosinophils	0	0–6.8
Basophils	0	0–1
Monocytes	2	2–7.6
Atypical lymphocytes	3	0
Metamyelocytes	2	0
Lymphocytes	5	34.4–62.8

Immune cell profile (%)	Value	Reference range (%)
T cells	65.7	56–75
B cells	31.3	14–33
NK cells	1.2	4–17
CD4 T cells	45.6	28–47
Memory CD4 T cells	14.9	15–41
CD8 T cells	17.4	16–30
Memory B cells	3.7	1.4–6.6

Ig	Value	Reference range
IgG (mg/dl)	1,140	633–1,280 (6–8 years)
IgG1 (mg/dl)	868	490–1,140 (>18 years)
IgG2 (mg/dl)	240	150–640 (>18 years)
IgG3 (mg/dl)	65.4	11–85 (>18 years)
IgG4 (mg/dl)	63.3	3–200 (>18 years)
IgA (mg/dl)	140	33–202 (6–8 years)
IgM (mg/dl)	54.1	48–207 (6–8 years)
IgE (IU/ml)	103	<100 (>18 years)

Laboratory values were obtained from the patient during the acute phase of infection, on day 3 of hospitalization. Reference ranges are age-matched pediatric values used by the clinical laboratory, and for Ig’s, the applicable age group for each reference range is indicated in parentheses; age-specific pediatric intervals were not available for all analytes. Percentages in the leukocyte differential count are expressed as a proportion of total leukocytes and percentages of lymphocyte subsets are expressed as a proportion of total lymphocytes. Hb, hemoglobin; RBC, red blood cells; Ht, hematocrit; PLT, platelets; WBC, white blood cells; CRP, C-reactive protein; WBC-DC, white blood cell differential count; Seg, segmented neutrophils; NK, natural killer.

### Neutralizing anti–IFN-ω IgG auto-Abs in a child with severe HAdV-B7 pneumonia

**Figure 2.**
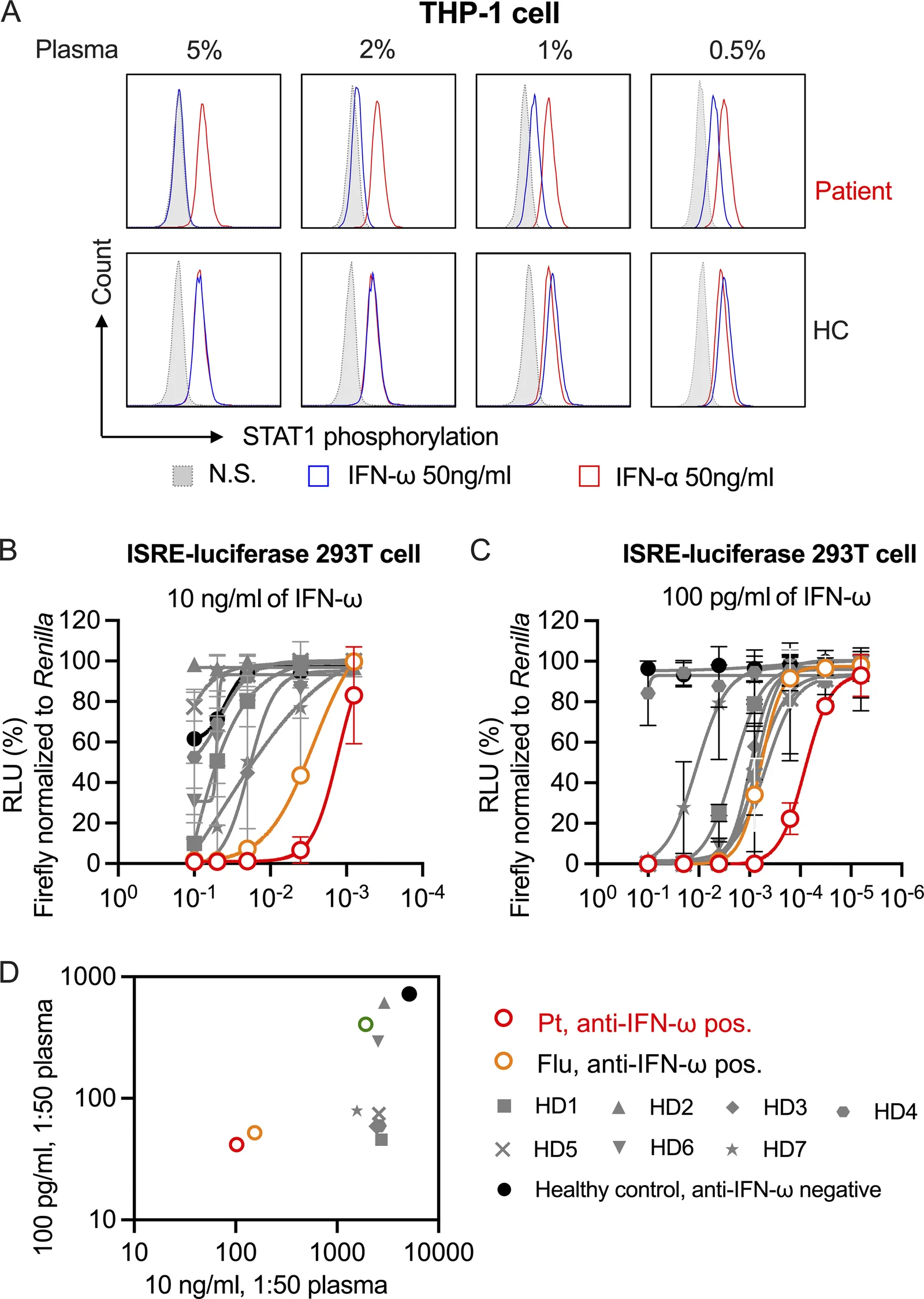
**Neutralizing activity of anti–IFN-ω auto-Abs in the patient’s plasma. (A)** Flow cytometry histograms showing IFN-α2– or IFN-ω–induced STAT1 phosphorylation in THP-1 cells in the presence of plasma from a healthy control or the patient. Plasma samples were tested at dilutions of 5, 2, 1, and 0.5%. **(B and C)** IFN-ω–responsive reporter activity (relative luminescence units, RLU; normalized to *Renilla* luciferase) in the presence of serial dilutions of plasma. Cells were stimulated with 10 ng/ml IFN-ω (B) or 100 pg/ml IFN-ω (C). **(B and C)** The data shown are the mean ± SD from three independent experiments (*n* = 3). **(D)** Summary of the potency of neutralization across samples. N.S., no stimulation; HD, healthy donor with anti–IFN-ω auto-Ab; Pt, the index patient; Flu, anti–IFN-ω auto-Ab–positive patient with H1N1 infection; HC, healthy control. The index patient’s plasma was collected during follow-up, 9 years after the initial infection.

**Figure S1.**
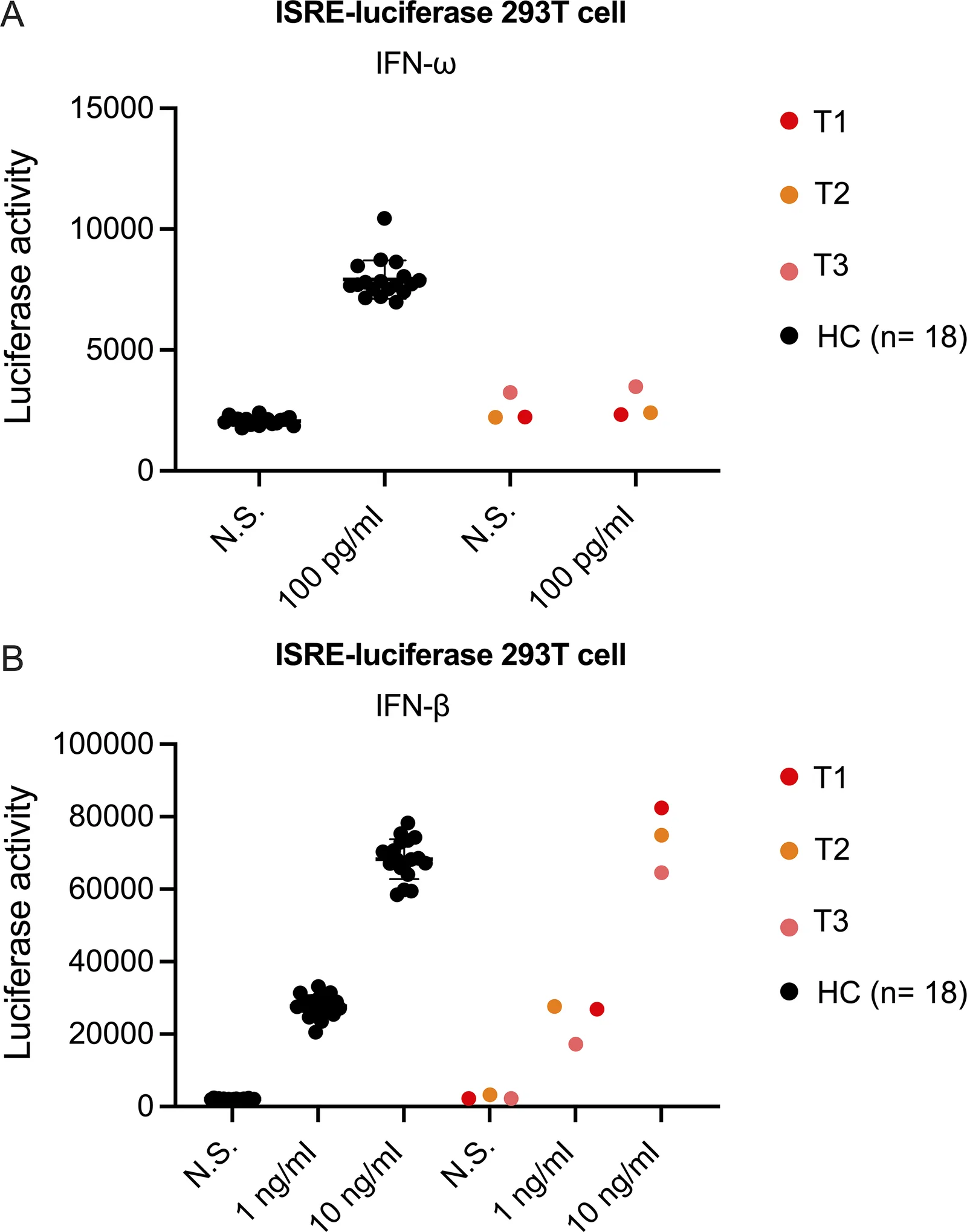
**The patient’s plasma persistently neutralizes IFN-ω. (A and B)** HEK293T cells stably expressing an ISRE reporter were stimulated with 5% patient plasma in combination with 100 pg/ml IFN-ω (A) or 1 or 10 ng/ml IFN-β (B) for 24 h. Patient plasma samples were collected at three time points (T1, T2, and T3), including the acute phase and after recovery. N.S., no stimulation; HC, healthy control.

Given the established contribution of AAN-I-IFNs to severe viral infections, we first screened plasma from children with adenoviral infection for auto-Abs against IFN-α and IFN-ω. We evaluated seven patients (Table 2), one of whom—the index case with severe HAdV-B7 pneumonia—harbored auto-Abs against IFN-ω but not IFN-α. Anti–IFN-ω auto-Abs were readily detectable during the acute phase and persisted after clinical recovery (Fig. 1 B). These findings prompted further investigations of the specificity and function of these antibodies in the context of severe adenoviral pneumonia. We characterized the Ig isotypes involved by performing ELISA, which demonstrated that the anti–IFN-ω auto-Abs were predominantly of the IgG subclass—mostly IgG1 and IgG3—with negligible levels of IgM, IgA, or IgE (Fig. 1, C and D). We then assessed the functional capacity of these auto-Abs by evaluating IFN-I–induced STAT1 phosphorylation in THP-1 cells. The patient’s plasma selectively inhibited IFN-ω–induced STAT1 phosphorylation in a dose-dependent manner, with 5% plasma completely abolishing the activity of 50 ng/ml IFN-ω, whereas IFN-α2–mediated signaling remained intact (Fig. 2 A). The patient therefore harbored highly selective neutralizing auto-Abs targeting IFN-ω. Previous population studies have reported the presence of AAN-I-IFNs in ∼2% of healthy children (0–18 years), with pediatric prevalence estimates of ∼2.0% for anti–IFN-ω (1.7% for low titer and only 0.35% for high titer to anti–IFN-ω), ∼0.2% for anti–IFN-α2, and ∼0.04% for anti–IFN-β auto-Abs (36, 37). In our pediatric cohort, we found that 7 of 455 (1.5%) healthy children carried anti–IFN-ω auto-Abs without anti–IFN-α auto-Abs (Table 2). We further evaluated the neutralizing activity of these plasma samples using an IFN-stimulated response element (ISRE) reporter assay. 10% plasma from five healthy children partially inhibited a low dose of IFN-ω (100 pg/ml), whereas three children’s plasma showed partial inhibition at a high dose (10 ng/ml) (Fig. 2, B and C). In contrast, plasma from the index patient and a previously described child with influenza-associated AAN-I-IFNs (32) robustly neutralized both doses of IFN-ω, with complete inhibition maintained even at 1% plasma dilution, and demonstrated markedly lower residual IFN-ω activity at 1:50 dilution compared with healthy controls (Fig. 2, B–D). Notably, the index patient’s plasma retained strong neutralizing ability against IFN-ω after follow-up (Fig. S1 A). Given prior reports of anti–IFN-β auto-Abs in a subset of patients with severe COVID-19, we further assessed the neutralizing activity of the patient’s plasma against IFN-β, using samples from the acute phase and after recovery, in an ISRE reporter assay. The patient’s plasma did not neutralize the activity of IFN-β at either 1 ng/ml or 10 ng/ml (Fig. S1 B), suggesting the absence of anti–IFN-β auto-Abs in the patient’s plasma. Collectively, these data demonstrate that the patient with severe HAdV-B7 pneumonia had strongly neutralizing anti–IFN-ω IgG auto-Abs. These findings highlight a potential pathogenic contribution of anti–IFN-ω auto-Abs to severe pediatric adenoviral infections.

**Table 2.** The prevalence of anti–IFN-ω auto-Abs in our cohort

​	Numbers	Number of patients positive for anti–IFN-ω auto-Abs	Frequency of positive for anti–IFN-ω auto-Abs
Healthy donors	455	7	7/455 (1.5%)
HAdV infections	7	1	1/7 (14.3%)

Plasma samples from healthy donors and from children with HAdV infection were screened for auto-Abs against IFN-ω by ELISA, as described in Materials and methods. The single positive patient in the adenovirus-infected group is the index case described in this study.

### Human peripheral blood mononuclear cells (PBMCs) are responsible for IFN-ω production during viral infections

**Figure 3.**
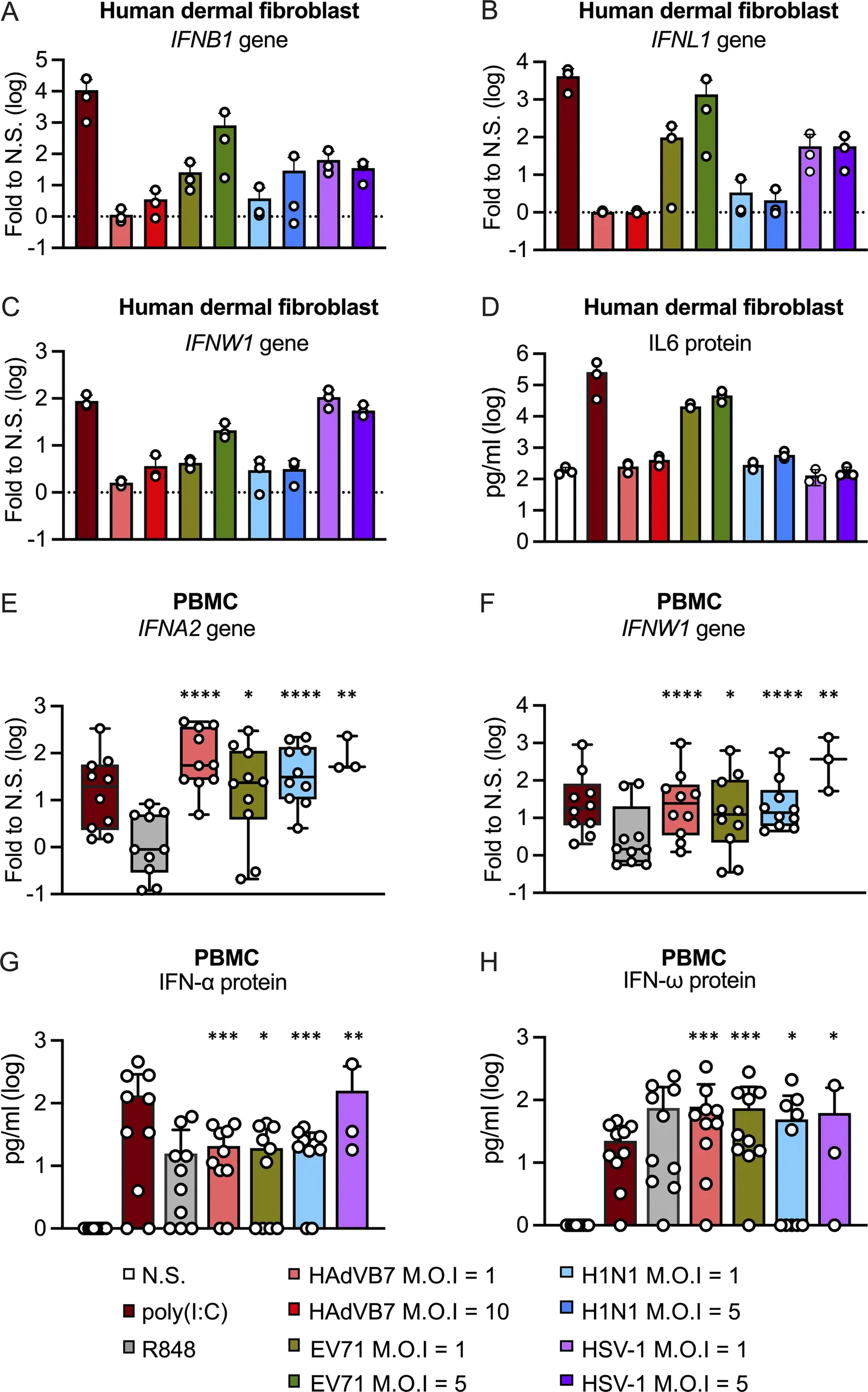
**IFN-ω is robustly induced in human PBMCs but not in fibroblasts. (A–C)** Human dermal fibroblasts were left unstimulated, stimulated with poly(I:C), or infected with HAdV-B7, EV71, H1N1, or HSV-1. Expression of the *IFNB1*, *IFNL1*, and *IFNW1* genes was quantified by qPCR at 24 h, with normalization against unstimulated controls. The results are presented on a logarithmic scale. **(D)** IL-6 protein levels were determined by ELISA at 48 h. The data shown are the mean ± SD of three independent experiments. **(E and F)** Human PBMCs were left unstimulated, stimulated with poly(I:C) or R848, or infected with HAdV-B7, EV71, H1N1, or HSV-1. Expression of the *IFNA2* and *IFNW1* genes was assessed by qPCR at 24 h, normalized against unstimulated controls, and the results are presented on a logarithmic scale. **(G and H**) IFN-α and IFN-ω protein levels were quantified by ELISA at 48 h. The data shown are the mean ± SD for 10 independent donors, except for HSV-1 (*n* = 3). Each dot indicates an individual donor. Statistical significance was determined by nonparametric Mann–Whitney tests based on ΔCt values. *P < 0.05; **P < 0.01; ***P < 0.001; and ****P < 0.0001. The dotted line indicates the baseline corresponding to unstimulated (N.S.) conditions (fold-change = 1). N.S., no stimulation; HSV-1, herpes simplex virus; M.O.I, multiplicity of infection.

**Figure S2.**
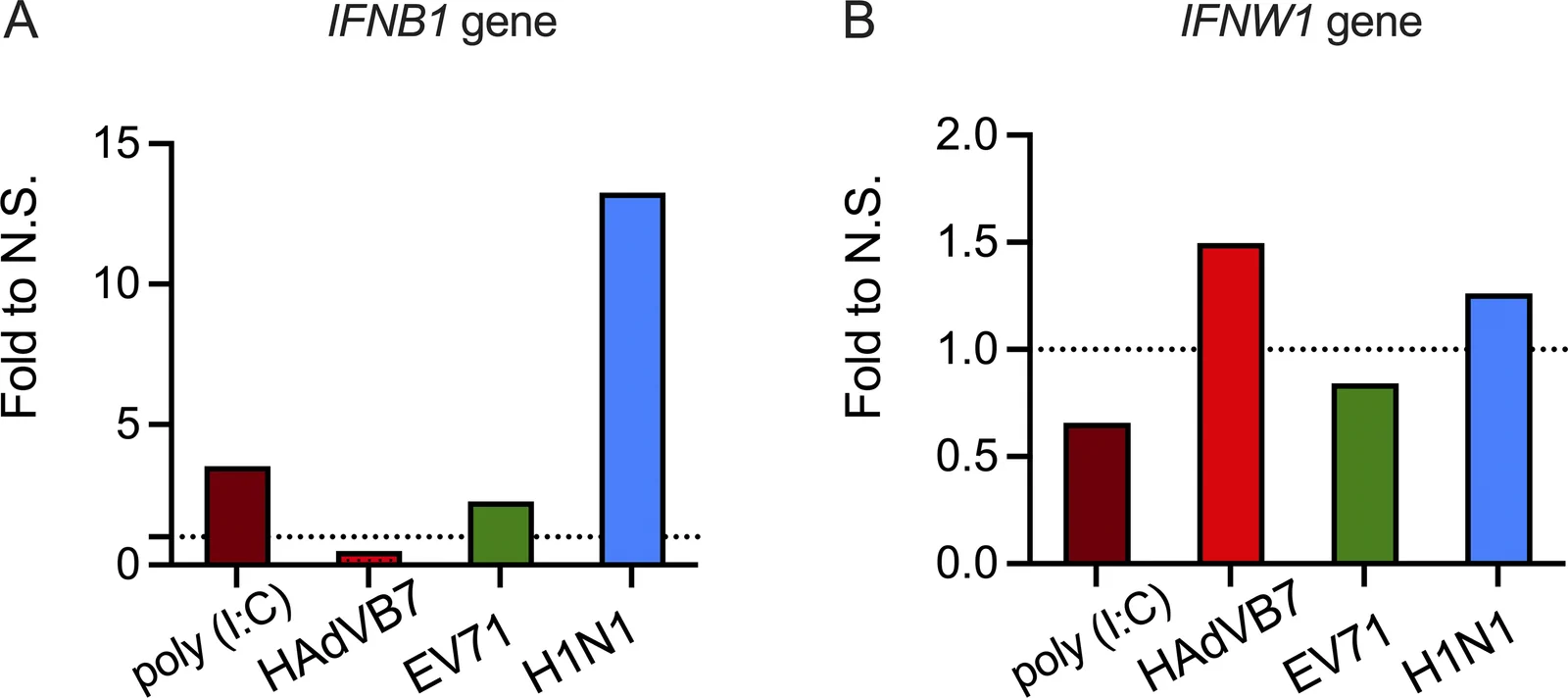
**TT1 lung epithelial cells do not produce IFN-ω upon viral stimulation.** **(A and B)** TT1 cells were left unstimulated (N.S.), stimulated with poly(I:C), or infected with HAdV-B7, EV71, or H1N1. Expression of the *IFNB1* (A) and *IFNW1* (B) genes was quantified by qPCR with normalization against unstimulated controls. The dotted line indicates the baseline (fold change = 1). N.S., no stimulation.

IFN-I responses, including those mediated by IFN-ω, have been shown to exert potent antiviral activity in animal models (23, 38). Given the recent identification of neutralizing auto-Abs against IFN-α and/or IFN-ω in patients with severe viral infections (30, 31, 32, 33, 34), we sought to determine whether viral pathogens induce endogenous IFN-ω production in human cells and to identify the cellular sources involved. We first studied human dermal fibroblasts stimulated with poly(I:C) or infected with HAdV-B7, enterovirus 71 (EV71), influenza A virus (H1N1), or HSV-1. Poly(I:C), EV71, and HSV-1 induced a robust transcriptional upregulation of *IFNB1*, *IFNL1*, and *IFNW1* at 24 h (Fig. 3, A–C). Despite this induction of transcription, the IFN-ω protein remained undetectable in fibroblast supernatants at both 24 and 48 h (data not shown). By contrast, IL-6 was readily produced under the same conditions, confirming effective cellular activation (Fig. 3 D). Consistently, TT1 lung epithelial cells displayed minimal *IFNW1* expression following stimulation with poly(I:C), HAdV-B7, EV71, or H1N1 (Fig. S2), indicating that epithelial cells make no substantial contribution to IFN-ω production during viral challenge. We then evaluated PBMCs, which are known to produce IFN-α. PBMCs were stimulated with the same panel of viral pathogens and with R848, a TLR7/8 agonist. All viral stimuli strongly upregulated *IFNA2* and *IFNW1* transcription (Fig. 3, E and F), with the detectable production of IFN-α and IFN-ω proteins at 48 h (Fig. 3, G and H). Together, these findings demonstrate that human PBMCs—but not fibroblasts or epithelial cells—are the principal producers of IFN-ω during viral infection, highlighting their central role in IFN-ω–mediated antiviral immunity.

### Undetectable circulating IFN-ω in the index patient plasma during infection

**Figure 4.**
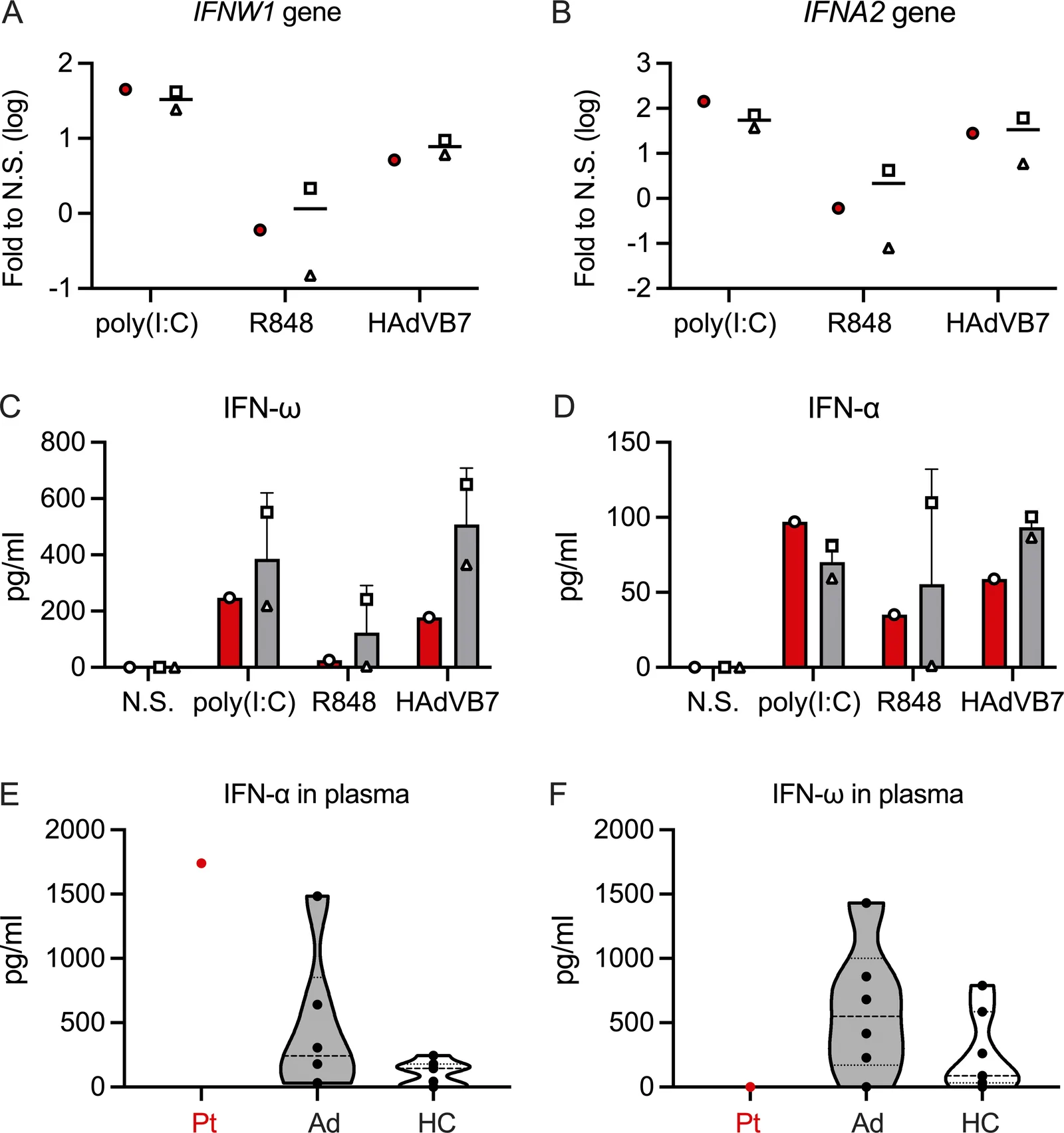
**Preserved PBMC IFN-I responses with no circulating IFN-ω in this patient. (** **A and B** **)** Patient PBMCs were left unstimulated, stimulated with poly(I:C) or R848, or infected with HAdV-B7 at an MOI of 2. *IFNW1* (A) and *IFNA2* (B) mRNA levels were assessed by qPCR at 24 h and are expressed as fold-changes relative to the unstimulated condition. **(C and D)** IFN-ω (C) and IFN-α (D) protein levels in culture supernatants were determined by ELISA at 48 h. The red circle indicates the index patient, and the square and triangle indicate two healthy controls. **(E and F)** IFN-α (E) and IFN-ω (F) concentrations in the plasma of adenovirus-infected children and healthy controls were determined by ELISA. Ad, adenovirus-infected children; HC, healthy control; Pt, patient. The index patient’s PBMCs in A–D were obtained from the patient during follow-up, 11 years after the initial infection. Plasma samples in E and F were collected during the acute phase of infection.

We investigated whether the patient’s circulating immune cells retained the ability to produce IFN-I by stimulating PBMCs from the patient and healthy controls with poly(I:C), R848, or HAdV-B7. The patient’s PBMCs displayed an induction of *IFNW1* and *IFNA2* transcription similar to that in healthy controls (Fig. 4, A–B) and they secreted both IFN-ω and IFN-α protein in response to each stimulus (Fig. 4, C and D). Thus, the patient’s PBMCs maintained normal upstream signaling capacity for IFN-I production. We then assessed circulating IFN-α and IFN-ω levels by ELISA in children with adenovirus infection, including the index patient. IFN-α was detectable in most acute plasma samples, with concentrations varying across patients. In contrast, IFN-ω was undetectable in the index patient (<4.7 pg/ml), despite being present in most adenovirus-infected controls (Fig. 4, E and F). Together, these data demonstrate that the index patient retained a normal cellular capacity for IFN-α and IFN-ω production, yet circulating IFN-ω was undetectable in vivo—consistent with possible sequestration or neutralization by anti–IFN-ω auto-Abs. These results suggest a model in which neutralizing anti–IFN-ω auto-Abs decrease the bioavailability of IFN-ω during adenoviral infection, potentially compromising IFN-ω–mediated antiviral defense.

### Anti–IFN-ω auto-Abs impair the antiviral effects of IFN-ω in vitro

**Figure S3.**
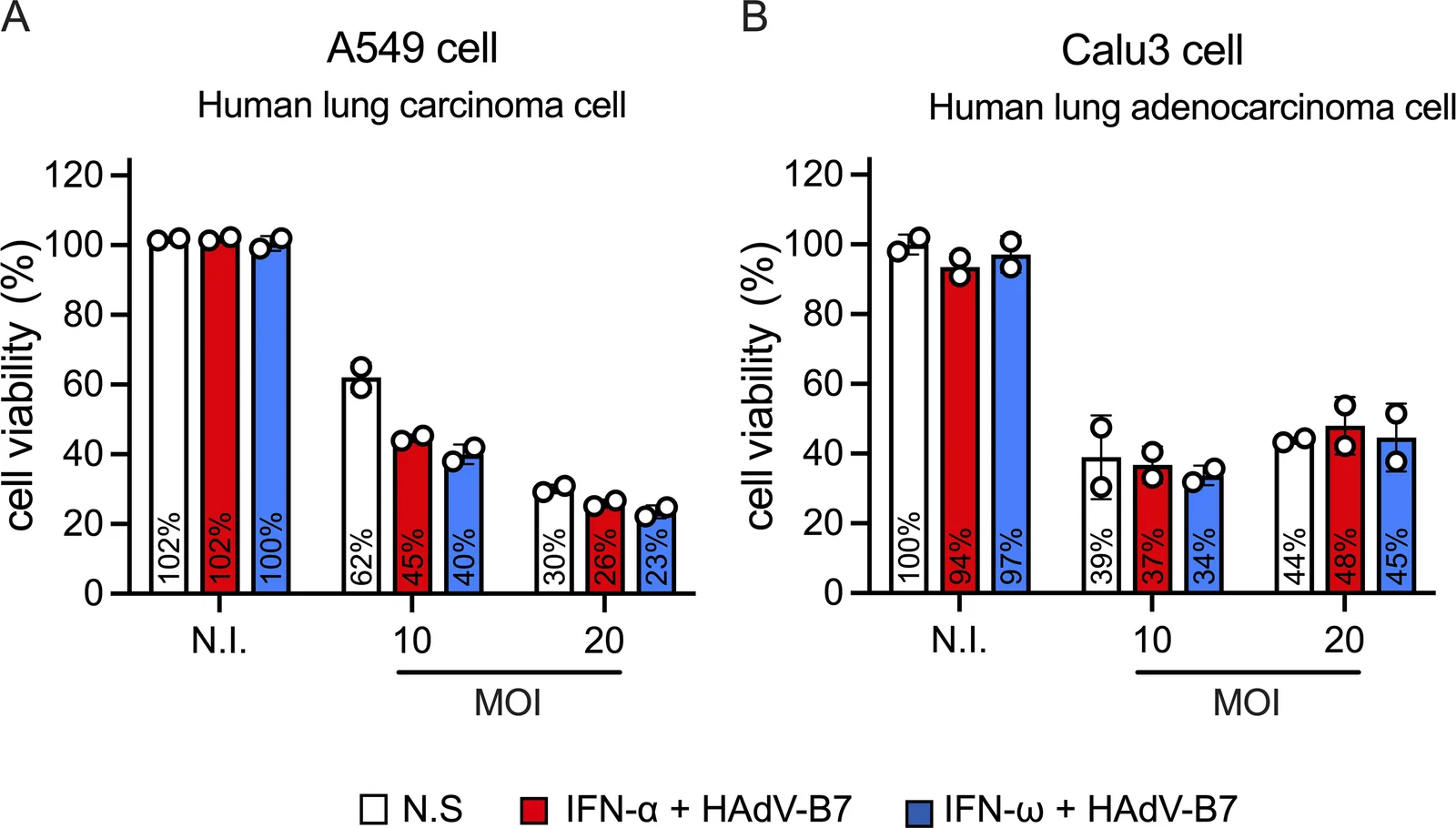
**IFN-I does not protect transformed lung epithelial cells from HAdV-B7–induced cytotoxicity. (A and B)** A549 (A) and Calu-3 (B) cells were left untreated or were incubated with IFN-α2 or IFN-ω for 24 h, and then infected with HAdV-B7 at an MOI of 10 or 20. At the indicated days after infection, virus-induced cytotoxicity was assessed in the CCK-8 assay. The data shown are means ± SD from two independent experiments performed in duplicate wells. N.I., no infection.

**Figure 5.**
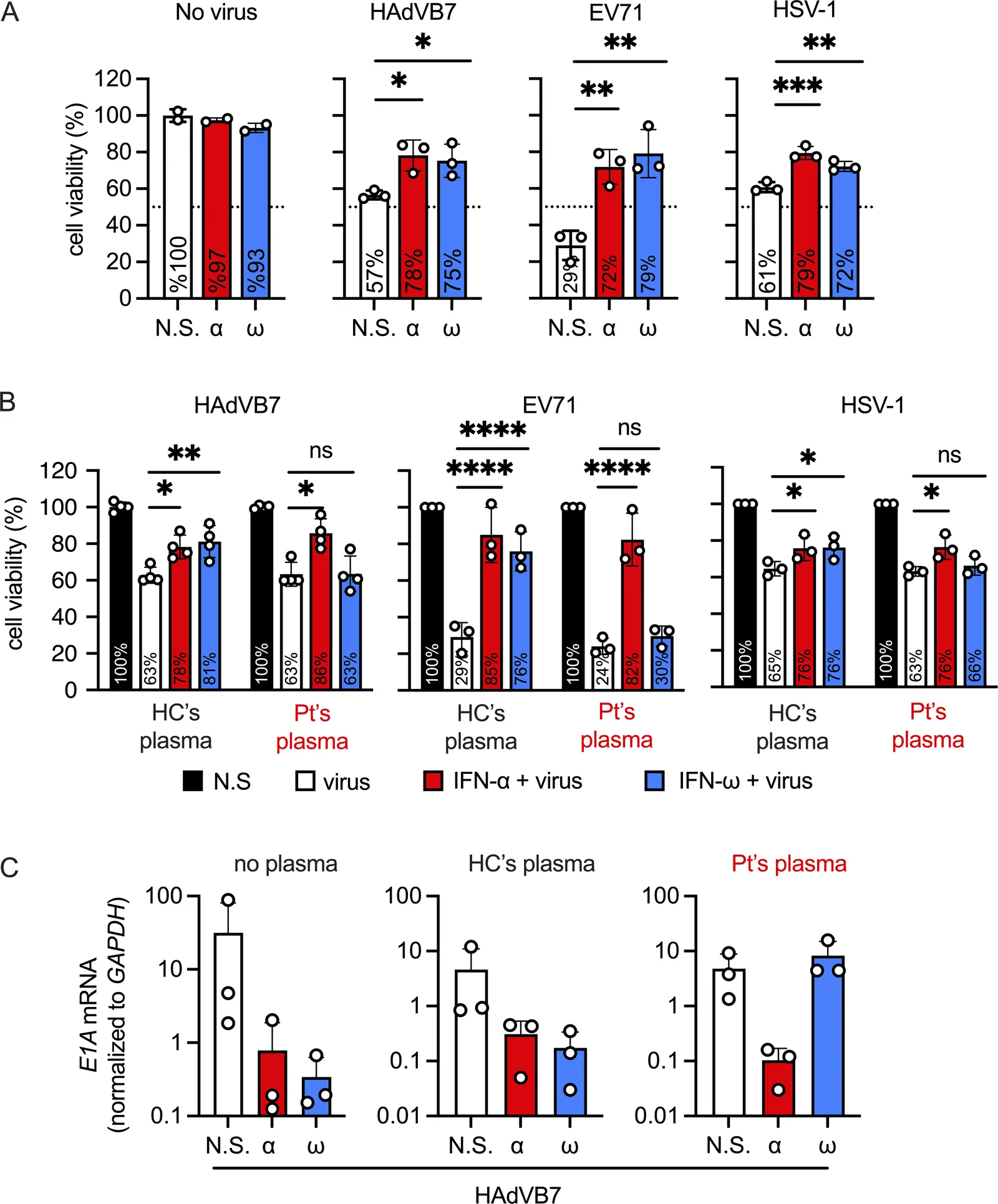
**Patient plasma abolishes IFN-ω–mediated antiviral activity. (A)** Human dermal fibroblasts (HDFs) were left untreated or were treated with IFN-α2 or IFN-ω and then infected with HAdV-B7, EV71, or HSV-1. Cell viability (virus-induced cytotoxicity) was quantified with the CCK-8 kit. **(B)** HDFs were treated with IFN-α2 or IFN-ω in the presence of 1% plasma from a healthy donor or the patient and infected as in A; cell viability was measured with the CCK-8 kit. **(C)** HDFs were treated with IFN-α2 or IFN-ω in the presence of plasma from a healthy donor or the patient and infected with HAdVB7; viral load was assessed by qPCR for E1A normalized against GAPDH. The data shown are means ± SD from three or four independent experiments. Differences were assessed by two-way ANOVA with Tukey's correction for multiple comparisons. ns, not significant; *P < 0.05, **P < 0.01, ***P < 0.001, and ****P < 0.0001. N.S., no stimulation; HC, healthy control; Pt, patient. Patient plasma was collected during follow-up, 9 years after the initial infection.

We investigated whether the anti–IFN-ω auto-Abs–containing plasma from the patient inhibited the antiviral activity of IFN-ω by first assessing the responsiveness of epithelial cell lines commonly used in adenovirus research. A549 lung carcinoma cells and Calu-3 lung adenocarcinoma cells were incubated with IFN-α2 or IFN-ω before HAdV-B7 infection. Neither IFN-I conferred measurable protection at a multiplicity of infection (MOI) of 10 or 20; virus-induced cytotoxicity remained similar in all treatment groups (Fig. S3). IFN-I therefore has minimal antiviral activity against wild-type adenovirus in transformed lung epithelial cells, consistent with prior observations that cancer-derived lines may have impaired IFN-I signaling or IFN-I–independent growth-inhibiting responses (39). We then studied primary human dermal fibroblasts, a physiologically relevant and permissive cell type for adenoviral replication. By contrast to what was observed for transformed epithelial cells, pretreatment of fibroblasts with IFN-α2 or IFN-ω significantly increased cell viability following viral infection. Specifically, IFN-α2 and IFN-ω increased cell viability by ∼20% following infection with HAdV-B7 (57–78% and 75%, respectively) and HSV-1 (61–79% and 72%, respectively), and by ∼40% following infection with EV71 (29–72% and 79%, respectively), whereas no survival benefit was observed in uninfected cells (Fig. 5 A). Thus, both IFN-α2 and IFN-ω provide antiviral protection in primary fibroblasts. We investigated whether anti–IFN-ω auto-Abs could abolish this antiviral activity by incubating primary fibroblasts with IFN-α2 or IFN-ω in the presence of 1% plasma from either the index patient or a healthy control. Healthy control plasma did not modify the protective effects of either interferon. By contrast, patient plasma selectively eliminated the antiviral activity of IFN-ω—while leaving IFN-α2–mediated protection intact—resulting in cell viability indistinguishable from that of untreated virus-infected controls. This selective neutralization was consistently observed across infections with HAdV-B7, EV71, and HSV-1 (Fig. 5 B). We assessed its impact on adenoviral replication directly by quantifying E1A mRNA levels to determine viral load. IFN-ω markedly reduced E1A expression in the presence of healthy control plasma but not in the presence of patient plasma, demonstrating a complete loss of antiviral activity (Fig. 5 C). By contrast, IFN-α2 decreased viral load regardless of the source of the plasma. Together, these results demonstrate that IFN-ω exerts antiviral activity against both DNA viruses (HAdV-B7, HSV-1) and RNA viruses (EV71) in primary fibroblasts. Crucially, the anti–IFN-ω auto-Abs present in the patient’s plasma selectively neutralize IFN-ω and abolish its protective antiviral function, supporting a pathogenic role for these auto-Abs during severe adenoviral infection.

## Discussion

We describe here a pediatric case of severe adenoviral pneumonia characterized by the presence of strongly neutralizing auto-Abs against IFN-ω. Adenoviral infections in children are typically self-limited, but this patient developed severe pneumonia and viremia due to HAdV-B7 despite normal clinical immunobiological findings and the absence of any known inborn error of immunity (IEI). Human PBMCs are the principal producers of IFN-ω during viral stimulation, and the patient’s PBMCs retained the ability to produce IFN-ω. However, ELISA revealed undetectable levels of IFN-ω in the patient’s plasma during acute infection, whereas IFN-α remained detectable, consistent with the presence of selectively neutralizing anti–IFN-ω auto-Abs in the circulation. Functional assays further demonstrated that the patient’s plasma abolished the antiviral activity of IFN-ω in vitro, impairing its ability to limit adenoviral replication and virus-induced cytotoxicity. Together, these findings support a pathogenic role for anti–IFN-ω auto-Abs in this case and raise the possibility that such antibodies could contribute to severe adenoviral disease in children.

Most adenovirus infections are mild and self-limiting, but a subset of these infections progress to severe disease due to a combination of host and viral factors (2). Host risks include immunosuppression (e.g., transplantation, corticosteroid therapy), extreme age (very young or very old), and underlying cardiopulmonary or neurologic disease (1, 5, 11). Viral determinants, such as high viral load, viremia, and virulent serotypes, such as HAdV-B7, further increase severity (12). In mice, the STING-mediated IFN-I pathway and STAT1 play a key role in controlling adenoviral challenge (40). In humans, adenovirus infection has been reported in IFNAR1 deficiency (41), and severe or disseminated disease has also been observed in autosomal recessive STAT1 deficiency, which compromises signaling downstream of IFN-I (42, 43). These findings not only illustrate the role of IFN-I in the control of adenovirus infections by human immunity but also raise the possibility that defects of this pathway, whether genetic or acquired via AAN-I-IFNs, confer a predisposition to severe adenoviral infection. This is the first case, to our knowledge, in which anti–IFN-ω auto-Abs have been implicated in impaired IFN-I responses leading to severe uncontrolled adenoviral pneumonia.

Our findings further support an underappreciated antiviral role for IFN-ω in humans. Although recombinant IFN-ω has long been used to treat viral infections in veterinary medicine (24), its physiological contribution to human antiviral immunity remains incompletely defined. Recent studies have shown that human monocytes and plasmacytoid dendritic cells are major sources of IFN-ω following cGAS- and CpG-mediated stimulation, respectively, consistent with the results of our PBMC stimulation assays. In addition, IFN-ω has been reported to exert potent antiviral activity in upper airway epithelial cells and to promote antiviral CD8^+^ T cell responses (44). Consistent with these observations, our in vitro experiments showed that IFN-ω conferred antiviral protection against both DNA viruses (HAdV-B7 and HSV-1) and the RNA virus EV71, with efficacy comparable with that of IFN-α. Together with recent reports linking anti–IFN-ω auto-Abs to severe outcomes in COVID-19, influenza, adverse reactions to yellow fever vaccination, West Nile virus infection, and other arthropod-borne viral diseases (30, 31, 32, 33, 34, 45, 46), these findings support a broader role for IFN-ω in human antiviral defense. More broadly, our data are consistent with the emerging concept that IFN-I subtypes are not fully redundant. Distinct IFN-I subtypes are differentially produced across cell types and tissues—for example, IFN-α by leukocytes, IFN-β by fibroblasts, IFN-ε in the female reproductive tract, and IFN-κ by keratinocytes—and can induce overlapping but nonequivalent interferon-stimulated gene (ISG) programs (16). Such specialization may provide complementary antiviral protection across different tissue environments and against distinct viral pathogens. Taken together, our results underscore the need to define more precisely the cooperative and compensatory interactions among IFN-α, IFN-ω, and other IFN-I during human viral infection.

AAN-I-IFNs have been reported in APS-1, *RAG* deficiency, systemic lupus erythematosus (47, 48, 49), and more recently in IEI affecting the alternative NF-κB pathway that disrupts medullary thymic epithelial cell maturation and reduces AIRE expression (50). Thymoma-associated AAN-I-IFNs have also provided evidence for aberrant thymic selection (51). In the context of COVID-19, AAN-I-IFNs occur in ∼4% of individuals over the age of 70 years, their prevalence increasing with age, suggesting an age-related immune dysregulation or breakdown of central and peripheral tolerance (36). In our patient, no defects were identified in genes regulating the alternative NF-κB/AIRE pathway. The mechanisms driving anti–IFN-ω auto-Ab production therefore remain to be determined. Previous studies have also reported strong associations between neutralizing anti–IFN-γ auto-Abs (nAIGAs) and specific HLA class II haplotypes, particularly HLA-DRB1*16:02/DQB1*05:02 and HLA-DRB1*15:02/DQB1*05:01, which are prevalent in Southeast Asian populations (52). Environmental triggers, such as *Aspergillus* exposure, may induce nAIGAs through molecular mimicry (53). Further investigations are required to determine whether similar genetic or environmental factors contribute to the emergence of anti–IFN-ω auto-Abs.

In summary, we report the first pediatric case of severe adenoviral pneumonia associated with strongly neutralizing anti–IFN-ω auto-Abs, revealing a previously unrecognized vulnerability of IFN-I–mediated antiviral immunity. These findings highlight the importance of IFN-ω in the early control of adenoviral infection and suggest that screening for anti–IFN-ω auto-Abs may help identify children at risk of severe adenoviral disease and other viral infections. The identification of additional cases will be essential to delineate the broader clinical impact of these auto-Abs and to guide potential therapeutic strategies targeting IFN-ω pathways.

## Materials and methods

### Cell culture

Primary human dermal fibroblasts were isolated from skin biopsy specimens from healthy donors. Fibroblasts, HEK293T, and Calu-3 cells were cultured in Dulbecco’s modified Eagle medium (Gibco) supplemented with 10% FBS and 1% penicillin–streptomycin (P/S). THP-1 cells were maintained in RPMI-1640 medium (Gibco) supplemented with 10% FBS and 1% P/S, and A549 cells were cultured in F-12K medium (Gibco) supplemented with 10% FBS and 1% P/S. All cells were incubated at 37°C in a humidified atmosphere containing 5% CO_2_.

### Human plasma and PBMC isolation

Peripheral venous blood from pediatric patients and healthy donors was collected into sterile heparinized tubes. Plasma was separated by centrifugation and stored at −80°C until use. PBMCs were isolated by density-gradient centrifugation on Ficoll-Paque (GE Healthcare) according to the manufacturer’s protocol.

### Detection of anti–IFN-ω auto-Abs

Anti–IFN-ω auto-Abs and their Ig isotypes were quantified by ELISA. Recombinant human IFN-ω (NBP2-35893; Novus Biologicals) was used to coat 96-well plates by incubation overnight at 4°C. Plates were washed, blocked, and incubated with plasma samples (1:100 dilution) for 2 h at room temperature. Bound antibodies were detected with horseradish peroxidase (HRP)–conjugated secondary antibodies specific for human IgG, IgM, IgA (Jackson ImmunoResearch), IgE (Invitrogen), and IgG subclasses (IgG1–4; Abcam). Following incubation with TMB substrate (BD Biosciences), absorbance at 450 nm was measured with a SpectraMax iD3 multimode reader (Molecular Devices). Results are expressed as optical density (OD) relative to control plasma.

### Intracellular cytokine staining

THP-1 cells were incubated with plasma from patients or controls and were then either left unstimulated or were stimulated with IFN-α2 (592706; BioLegend) or IFN-ω (BMS304; eBioscience) for 45 min at 37°C. Cells were fixed with BD Cytofix (554655; BD Biosciences), permeabilized with ice-cold methanol, and stained by incubation with anti–phospho-STAT1-PE (612564; BD Biosciences) for 45 min at room temperature in the dark. Samples were acquired on a BD FACSVerse flow cytometer and analyzed with FlowJo software.

### Luciferase reporter assay

HEK293T cells were cotransfected with an *ISRE*-driven firefly luciferase reporter plasmid and the pRL-SV40 *Renilla* vector, with X-tremeGENE 9 (6365779001; Sigma-Aldrich). The ISRE reporter was provided by Dr. Ming-Hong Lin (Kaohsiung Medical University, Kaohsiung, Taiwan), and pRL-SV40 was provided by Dr. Jean-Laurent Casanova (Rockefeller University, New York, NY, USA). After 24 h, cells were incubated with patient or control plasma and stimulated with IFN-ω (100 pg/ml or 10 ng/ml) for an additional 24 h at 37°C. Firefly and *Renilla* luciferase activities were quantified with the Dual-Glo Luciferase Assay System (E2920; Promega). Firefly luciferase values were normalized against *Renilla* luciferase and unstimulated control values and are expressed as a percent ISRE activity.

### Quantitative PCR (qPCR)

Human dermal fibroblasts were stimulated with poly(I:C) (27473201; Cytiva; 25 μg/ml) or infected with HAdV-B7, EV71, HSV-1, or H1N1 for 24 h. PBMCs were stimulated with the same stimuli or with R848 (tlrl-r848; InvivoGen; 1 μg/ml). Total RNA was extracted with the Quick-RNA MicroPrep Kit (R1050; Zymo Research), and cDNA was synthesized with the SuperScript III First-Strand Synthesis SuperMix (18080400; Invitrogen). qPCR was performed with TaqMan Gene Expression Assays (Applied Biosystems) for *IFNB1* (Hs01077958_s1), *IFNW1* (Hs00958789_s1), *IFNL1* (Hs00601677_g1), and *IFNA2* (Hs00265052). Reactions were run on a QuantStudio 3 system (Applied Biosystems), and gene expression levels were normalized against GAPDH by the 2^−ΔΔCq method.

### Cytokine ELISA

Concentrations of IFN-ω (NBP1-91178; Novus Biologicals), IFN-α2 (3424-1H-6; Mabtech), and IL-6 (555220; BD Biosciences) were determined in plasma and culture supernatants by ELISA, according to the manufacturers’ instructions.

### Viruses

Laboratory strains of HAdV-B7, EV71 (strain 2203), HINI (strain SWH1), and HSV-1 (strain 1878) were obtained from the Virology Laboratory of Chang Gung Memorial Hospital. Viral stocks were propagated in A549, human rhabdomyosarcoma (RD), Vero, or Madin-Darby canine kidney (MDCK) cells, respectively, and infectious titers were determined in standard plaque assays.

### Cell viability assay

Virus-induced cytotoxicity in human dermal fibroblasts was assessed with the Cell Counting Kit-8 (CCK-8; 96229; Sigma-Aldrich). Cells were seeded in 96-well plates and were treated with 1% patient or control plasma, with or without IFN-ω or IFN-α (5 ng/ml) for 24 h at 37°C, before viral infection. At the indicated times after infection, CCK-8 reagent was added, and absorbance at 450 nm was measured on a SpectraMax iD3 reader. Cell viability was calculated relative to uninfected controls and is expressed as a percentage.

### Quantification of HAdV-B7 E1A gene by qPCR

Fibroblasts were pretreated with 1% patient or control plasma, with or without IFN-ω or IFN-α (25 ng/ml) for 24 h at 37°C, and infected with HAdV-B7. Viral nucleic acids were extracted with the Quick-DNA/RNA Viral Kit (D7020; Zymo Research). The adenoviral E1A gene and GAPDH reference gene were quantified by SYBR Green qPCR, and relative E1A levels were obtained by normalization against GAPDH by the 2^−ΔCq method.

### Statistical analysis

Statistical analyses were performed with GraphPad Prism 9. Data are presented as the mean ± SD unless otherwise specified. Two-tailed Mann–Whitney U tests were used for comparisons between two groups. For experiments with two independent variables, two-way ANOVA was performed with Tukey’s correction for multiple comparisons. The number of independent experiments or biological replicates (*n*) is indicated in each figure legend. A P value <0.05 was considered statistically significant (*P < 0.05; **P < 0.01; ***P < 0.001; ****P < 0.0001).

### Online supplemental material

Fig. S1 shows that the neutralizing activity of the patient’s plasma against IFN-ω was maintained at follow-up and that the plasma did not neutralize IFN-β. Fig. S2 shows that TT1 lung epithelial cells do not upregulate IFNW1 following viral stimulation. Fig. S3 shows that neither IFN-α2 nor IFN-ω protects A549 or Calu-3 cells against HAdV-B7–induced cytotoxicity.

## Ethics statement

### Ethics approval

The study was approved by the Institutional Review Board of Chang Gung Memorial Hospital (IRB number: 201100792B0, 202500725B0A3, and 201001671B0).

### Consent to participate

Written informed consent was obtained from all family members, in accordance with the Declaration of Helsinki and the protocol approved by the institutional review board of Chang Gung Memorial Hospital. Blood samples and skin biopsy specimens were collected from the children and their relatives in accordance with this protocol.

### Consent for publication

Written informed consent for publication was obtained from the patient’s legal guardians.

## Data Availability

All data supporting the findings of this study are available within the article and its supplementary information files. Individual-level clinical data are not publicly available due to patient privacy but are available from the corresponding author (clku@cgu.edu.tw) upon reasonable request.
